# Stock Price Change Rate Prediction by Utilizing Social Network Activities

**DOI:** 10.1155/2014/861641

**Published:** 2014-03-25

**Authors:** Shangkun Deng, Takashi Mitsubuchi, Akito Sakurai

**Affiliations:** Graduate School of Science and Technology, Keio University, 3-14-1 Hiyoshi, Kohoku-ku, Yokohama 223-8522, Japan

## Abstract

Predicting stock price change rates for providing valuable information to investors is a challenging task. Individual participants may express their opinions in social network service (SNS) before or after their transactions in the market; we hypothesize that stock price change rate is better predicted by a function of social network service activities and technical indicators than by a function of just stock market activities. The hypothesis is tested by accuracy of predictions as well as performance of simulated trading because success or failure of prediction is better measured by profits or losses the investors gain or suffer. In this paper, we propose a hybrid model that combines multiple kernel learning (MKL) and genetic algorithm (GA). MKL is adopted to optimize the stock price change rate prediction models that are expressed in a multiple kernel linear function of different types of features extracted from different sources. GA is used to optimize the trading rules used in the simulated trading by fusing the return predictions and values of three well-known overbought and oversold technical indicators. Accumulated return and Sharpe ratio were used to test the goodness of performance of the simulated trading. Experimental results show that our proposed model performed better than other models including ones using state of the art techniques.

## 1. Introduction

Many business practitioners and researchers have developed various kinds of models to predict and analyze stock prices. For example, there are many studies that estimate and predict the stock prices and stock volatility by using historical stock prices or volumes data. Researchers such as Murphy [1] and Neely [2] showed that technical analysis is one possible way to predict the stock markets and foreign exchange markets successfully. Deng and Sakurai [3] used a single technical indicator from multiple timeframes to generate trading rules.

In the last decade, numerous researchers have used machine learning technologies, such as artificial neural network (ANN), support vector machine (SVM), genetic algorithm (GA), or some integrated models of these to predict stock price or exchange rate changes or to find trading rules for the stock or foreign exchange rate trading, by mining historical price or transaction volume data. For example, Hann and Steurer [4] and Chen and Leung [5] applied ANNs to foreign exchange rate prediction and found that the ANN-based model outperformed the linear models. Hadavandi et al. [6] proposed an ANNs-based integrated system for stock price forecasting. Kwok [7] and Cao [8] applied an SVM to predict the stock price and obtained good results. Shioda et al. [9] predicted the foreign exchange market states with SVM. de la Fuente et al. [10] and Allen and Karjalainen [11] applied GA to generate trading rules. Chien and Chen [12] applied a GA-based model to mine associative classification rules with stock trading data. Hirabayashi et al. [13] applied GA to finding trading rules for foreign exchange intraday trading by mining features from several technical indicators. Deng et al. [14] forecasted short term foreign exchange rates by a GA-based hybrid model. Their proposed models obtained better performance than that of some conventional models; however, they utilized the features extracted from only the historical prices or transaction volumes.

Other than stock time series data such as stock prices and transaction volumes, human factors have been considered recently as having significant impacts on the movements of stock prices. We hypothesize that these impacts could be quantified by looking at the Internet, since the advent of the digital information age has led many organizations and people to post news and their comments on the news on well-known social networks such as Twitter, Facebook, or Engadget. Therefore, by analyzing the dynamics of news items or user comments about relevant companies on the Internet, we may mine interesting possible correlations between social network activities with stock price movements. Previous researchers, such as Gruhl et al. [15], found correlations between sales rank and blog mentions. Mondria et al. [16] used Internet to search query data. Smith [17] used Google Internet search activity to predict volatility in the foreign currency. de Choudhury et al. [18] and Li et al. [19] modeled dynamics in social networks. de Choudhury et al. [20] identified several contextual properties of communication and described dynamics in user comments and used an SVM framework to learn and to predict stock prices, while Bollen et al. [21] used Twitter tweets to gauge the mood of stock markets and predict the stock market. Good performances on out-of-sample data of certain stocks showed that mining information from the Internet could be an alternative or complementary approach to prediction of changes in stock prices.

In this research, since the stock price movements are accumulation of individual behaviors which may appear as activities in social network service (SNS), we model stock price change rates as a function of quantitative features of news and comments in SNS and features relating to technical indicators of stock prices and trading volumes. To incorporate different types of features such as those for news and for prices into a regression model, a method called multiple kernel learning (MKL) by Bach et al. [22] is a very promising way. A strong point of MKL is that it allows us to combine different kernels when the job at hand requires one to use different kernels for different input features. In addition, MKL mitigates the risk of erroneous kernel selection to some degree, by taking a set of kernels and deriving a weight for each kernel, such that predictions are made based on the weighted sum of the kernels. Some researchers have applied MKL to predict foreign exchange rate or stock prices. For example, Fletcher et al. [23] applied MKL to the limit order book for predicting the movement and trading of the EUR/USD currency pair. Luss and d'Aspremont [24] applied MKL towards the prediction of abnormal returns from historical stock prices data and news. Lee et al. [25] applied MKL to the prediction of prices in Taiwan's stock market, obtaining results that surpassed those of some conventional methods. Good performances in these literatures inspired us to use MKL to utilize the information from different sources.

Evaluation measures are very important to evaluate the performances of models. The root mean square error (RMSE) is a measure which is often used for evaluating prediction results. However, given that people will sell or buy stocks when they can predict the stock price, the goodness of the predictions cannot be provided by differences of prediction and real values alone; a proper measure should be the trading profits based on the prediction. In addition, beyond the accumulated returns, most investors also pay close attention to the variability of returns. In other words, they hope the proposed model can increase profits as well as decrease associated risks while doing so. Therefore, to evaluate the appropriateness of prediction, we should not confine ourselves to RMSE and we should also use accumulated returns and returns to variability ratio or Sharpe ratio that further considers risk free profits.

To evaluate appropriateness of predictions by the accumulated returns and returns to variability ratio (or Sharpe ratio), simulated trading should be conducted based on the prediction, which requires us to define a trading rule. The trading rule is a set of rules that specify under what condition an action “sell,” “buy,” or “no trade” should be taken. Since the conditions include some parameters which should be set according current market situations, they should be learned from data. We did not consider transaction costs in this paper since the purpose of this research is to evaluate the prediction performance of the proposed model, not trading. There would be a case that a model could attain higher than 50% hit ratio of predicting directions; however, it is possible that this model's prediction is correct for smaller magnitude of movements but incorrect for larger magnitude of movements, which is not favorable for application of the model to real financial markets. We want to show that our proposed model yields positive returns and therefore the case does not happen in our proposed model.

For learning the trading rules, we adopted GA with the resulting accumulated profits as the fitness value of chromosomes, because the profit is generated through transactions that are discrete events. As demonstrated in Allen and Karjalainen [11], GA is a good method for finding good trading rules.

In summary, our study makes three main contributions. First, we extract features from both the time series data source and a social network source, in contrast to previous studies (e.g., de Choudhury et al. [20]) which considered properties of social network to predict stock price movements, and Luss and d'Aspremont [24] used text data of news for the prediction of abnormal returns. These literatures inspired us to attempt the prediction of stock prices by extracting features from time series data and a social network.

Second, we use the multiple kernel regression (MKR) framework to optimally combine the features of time series data, news, and user comments, in contrast to other works (e.g., de Choudhury et al. [20]) which used a single kernel for the SVM. Results from Hann and Steurer [4], Chen and Leung [5], Kwok [7], and Cao [8] prove that ANNs and SVMs (especially the latter) are good models to predict stock price change rates. However, given that the input features extracted from the time series of historical stock price change rates and those from a social network have different properties, we should consider using different kernels for input feature sets from different types of sources. However, it is not easy to assign good kernels manually. Therefore, we use MKL to solve this problem.

Third, for generating trading signals, we use not only the predicted stock price change rates from the MKL model, but also three well-known overbought and oversold technical indicators. In addition, we consider thresholds over which the difference between the predicted value and current value should prompt an action: we buy if the combined decision value is greater than the buying threshold, and we sell if the combined decision value is less than the selling threshold. The best values of both thresholds are learned in the GA learning periods.

The rest of this paper is arranged as follows.  Section 2 describes the methodology for this research. Technical indicators and input features we extract from stock historical traded data and the social network are described in Section 3.  Section 4 describes the structure of the proposed model. The experiment design is described in Section 5. Baseline and other models for comparative experiments and the evaluation measures are described in Section 6. The experimental results and discussions pertaining to them are reported in Section 7. Finally, Section 8 concludes this study.

## 2. Methodology

In this section, we first introduce support vector regression and multiple kernel regression. Thereafter, we present GA procedures.

### 2.1. Support Vector Regression (SVR)

SVR is a version of the SVM [26]. SVR, as SVM, estimates a linear function defined in a feature space of high dimension and is renowned for its ability to perform well when there are many relevant and irrelevant features.

Generally, in a regression problem, suppose we are given a set of training examples {(*x*_1_, *y*_1_), (*x*_2_, *y*_2_), (*x*_3_, *y*_3_),…, (*x*_*l*_, *y*_*l*_)}, where *x*_*i*_ ∈ *R* ^*n*^,  *y*_*i*_ ∈ *R*,   *i* = 1,2,…, *l*, and each *y*_*i*_ is the output value for the input vector *x*_*i*_; a regression model is learned from these patterns and used to predict the target values. SVR solves a minimization problem by balancing the empirical error and a regularization term, where the error is measured by Vapnik's  *ε*-insensitive loss function as follows:

(1) $$\begin{array}{c} \underset{w}{\min}\frac{1}{2}{||w||}^{2}+C\frac{1}{l}\overset{l}{\underset{i=1}{\sum }}L_{\varepsilon },\\ L_{\varepsilon }=\{\begin{array}{cc} \left|y_{i}-w*\Phi \left(x_{i}\right)-b\right|-\varepsilon & \text{if}\;\;\left|y_{i}-w*\Phi \left(x_{i}\right)-b\right|\geq \varepsilon \\ 0 & \text{otherwise}, \end{array} \end{array}$$

where *w* and *b* are a weight vector and an offset, respectively, which define the maximum margin hyperplane. *l* is the number of training samples, *ε* is the allowed precision, and *C* parameter is used for trade-off between model complexity and training error. Note that Φ(·) is a possibly nonlinear mapping from the input space to a feature space.

### 2.2. Multiple Kernel Regression (MKR)

A conventional SVR is applied to a single feature type. In this study, we use MKR, which is a regression version of MKL, to integrate the features from different information sources. In MKR, we train an SVR with an adaptively weighted combination of kernels that combine different kinds of features. The combined kernel is as follows:

(2) Kcomb(x,y)=∑j=1KβjKj(x,y) with  βj≥0, ∑j=1Kβj=1, 

where *β*_*j*_ are weights to combine subkernels *K*_*j*_(*x*, *y*). MKR will estimate the optimal weights from the training data. By preparing one subkernel for each feature set and estimating weights by MKL, we obtain an optimal combined kernel.

Sonnenburg et al. [27] proposed an efficient MKR algorithm to estimate optimal weights and SVR parameters simultaneously by iterating the training steps of a conventional SVM. In our experiments, we used the MKR library included in the Shogun toolbox.

### 2.3. Genetic Algorithm (GA)

Goldberg [28] provided an excellent discussion on the use of GAs for solving optimization problems. GAs start with an initial set of feasible solutions, called a “population.” The individual solutions in the population are known as chromosomes. Each chromosome, in turn, is made up of a number of genes that encode representations of a part of the solution. In every iteration (referred to as a “generation” in GA terminology), the current population evolves using reproduction strategies such as crossover and mutation. A fitness function is used to evaluate the chromosomes, and the survival of a chromosome from one generation to the next is biased in favor of the fittest chromosomes. In addition to reproduction strategies, an elitist strategy can be used to propagate the fittest chromosomes to the next generation. A combination of these strategies helps the population improve from generation to generation until the fittest member of the population represents a near optimal solution.

Steps 1 to 6 show the procedures of the GA based on Goldberg [28].

**Step 1 (initialization)**

Step 1 generates the initial population.

**Step 2 (evaluation)**

After the initialization step, each chromosome is evaluated using a fitness function.

**Step 3 (selection)**

Selection is a process in which suitable chromosomes are chosen from the parent population for the next generation. In this step, we adopt the tournament selection procedure. This step is repeated until the number of chromosomes selected is equal to the size of the population. In order to ensure the propagation of elite chromosomes, this model uses the Elitism Mechanism. This mechanism selects *P*% individuals, which have the relatively best fitness values, to be the offspring in the next generation, while the remaining individuals go through the genetic operations.

**Step 4 (crossover and mutation)**

Crossover operates by swapping the corresponding segments of a string representation of the parents and extends the search for a new solution. Mutation is a genetic mechanism. It randomly chooses a member of the population and changes one randomly chosen bit in its bit string representation.

**Step 5 (evaluation)**

Each chromosome is evaluated using the designed fitness function.

**Step 6 (examination of termination criteria)**

Steps 2 to 5 are repeated until the termination criteria are satisfied. The proposed algorithm is terminated if the maximum number of generations is achieved or if a solution with the highest fitness has remained unchanged for several generations.

## 3. Technical Indicators and Features

### 3.1. Technical Indicators

There are numerous influential trading technical indicators that are widely recognized and used by traders around the world. Some technical indicators are fairly straight forward to obtain and have proven to be successful in trading history. Among them, technical indicators such as the moving average (MA), rate of change (ROC), and moving average convergence and divergence (MACD) help traders to spot or follow trends, while the bias ratio (BIAS), Williams %*R* (WPR), and relative strength index (RSI) are used for identifying overbought and oversold conditions of a stock.

Table 1 shows the list of technical indicators used in this research; Price(*k*) refers to the closing price at time period *k*; *n* is the length of timeframe to calculate values of indicator. Note that the indicators are applied to any time series including trading volume.

The MA is used to understand the present trend and, thus, is a so-called trend-following index. It is used to emphasize the direction of a trend and to smooth out price fluctuations which are just random. The simple moving average (SMA) is a simple mean value with identical weights used for past prices, while the exponential moving average (EMA) is the average value of prices of a stock for a given length of timeframe, attributing greater weight to newer changes and less weight to older ones.

The MACD is intended to predict changes in the market tendency. It provides two indicators: MACD and the MACD signal. MACD shows the difference between a fast and slow EMA of closing prices. “Fast” refers to a short-period average and “slow” a long-period average. When MACD (*t*) is greater than 0, the short and steep uptrend is more influential than the long and gentle uptrend, which means that the stock price is likely to go up in the near future. Based on the default parameters, MACD is the difference between the 12-period and 26-period EMAs. The default values (12, 26, and 9) of MACD parameters can be changed based on the needs of the traders. In our research, we simply used the default values of MACD parameters because this value set is widely recognized and used in the world.

The rate of change (ROC) is a technical indicator showing the change rate between today's closing price and the closing price *N* days ago. We set *N* = 1 in this research for prediction on stock price changes one-day ahead.

The BIAS, WPR, and RSI are usually used to judge whether the stock is considered to be in possible oversold, overbought, or normal conditions. An extremely high or low value is a signal to the trader to buy when the stock is oversold and to sell when it is overbought. The parameter *n* of these indicators could be set by the traders. In this research, we use GA for obtaining the best parameter *n* of these indicators in the training period.

### 3.2. Features

Features used for proposed model are from three sources: historical traded prices and volumes of stocks, news in social network service, and user comments on the news.

#### 3.2.1. Features from Historical Prices and Volumes

The ROC, SMA, and MACD are often used to understand the present trend by traders. Therefore, we use these technical indicators features for historical prices and transaction volumes, which are shown in Table 2.

#### 3.2.2. Features from News and User Comments

Table 3 shows a list of numerical features from news items. The greater the number of news items about a certain company, the more the impact of the event described by that news item on a future event. Hence, the first numerical feature is the number of news per day *f*_*t*_^*c*^, where *t* is the date and *c* is the stock described in the news. In addition, the SMA is used to understand the present trend; thus SMA of frequency of news (FNSMA) is used as feature. We set the parameter *n* for SMA to be 7 because we assume that the return of a company stock on a certain weekday depends on its stock prices and communication activity pertaining to it in the preceding 7 days.

Table 4 shows a list of numerical features from user comments which are defined similarly to news.

The number of comments in all the posts per day *F*_*t*_^*c*^ is extracted from user comments, where *t* is the date and *c* is the stock of the company mentioned in the comments. The definition of SMA of frequency of comments (FCSMA) is shown in Table 4.

In addition, the longer the comment on a news item pertaining to a certain company, the more attention users are bound to focus on that company. Hence, we calculate the average and standard deviation of the length of user comments. Their definitions are shown in Table 4, where *a*_*t*_^*c*^ is the average length of comments about stock *c* on date *t* and *b*_*t*_^*c*^ is the standard deviation of the length of comments on date *t*. The total number of comments on date *t* is *m* and *l*_*k*_^*c*^ is the length of a comment about stock *c*.

## 4. Proposed Model

Our model first makes prediction of stock price change rates by multiplying kernelized linear function and then infers trading position to take, that is, sell, buy, or retreat for a while, by a linear function of the predicted value and technical indicators. The former regression function is learnt by MKR and the latter prediction function is learnt by GA. The model is expressed by the following:

(3) pred(x1,x2,y1)=〈u,ϕ(x1,x2,y1)〉−b,

(4) TDV(x1,x2,y1,y2,y3,y4)  =〈w,(pred(x1,x2,y1),y2,y3,y4)〉,

(5) S(x1,x2,y1,y2,y3,y4)  ={+1if  TDV(x1,x2,y1,y2,y3,y4)>θbuy−1if  TDV(x1,x2,y1,y2,y3,y4)<θsell0otherwise,

where *u* and *w* are weight vectors and *b* is an offset. *x*_1_, *x*_2_, and *y*_1_ are vectors of features from news, user comments, and historical trading data, respectively. (pred(*x*_1_, *x*_2_, *y*_1_), *y*_2_, *y*_3_, *y*_4_) is a vector of four elements: prediction of stock price change rate and RSI, BIAS, and WPR of historical prices. 〈 〉 means the dot product, and *ϕ*( ) is the function that maps inputs to higher dimensional feature space and that accompanies a kernel function.

Note that pred in (3) is a regression function for prediction. TDV, which stands for trading decision value, is a function to combine values of MKR prediction and values of three overbought and oversold indicators. *S* is the function that outputs trading signal. As a trading signal, *S*(*x*_1_, *x*_2_, *y*_1_, *y*_2_, *y*_3_, *y*_4_) = +1 designates “buy,” *S*(*x*_1_, *x*_2_, *y*_1_, *y*_2_, *y*_3_, *y*_4_) = −1 designates “sell,” and *S*(*x*_1_, *x*_2_, *y*_1_, *y*_2_, *y*_3_, *y*_4_) = 0 designates “no trade.” The features *x*_1_ and *x*_2_ are those from SNS, and *y*_1_ to *y*_4_ are from historical trading data. The threshold values *θ*_buy_ and *θ*_sell_ will be described in Section 4.3.

**Figure 1:**
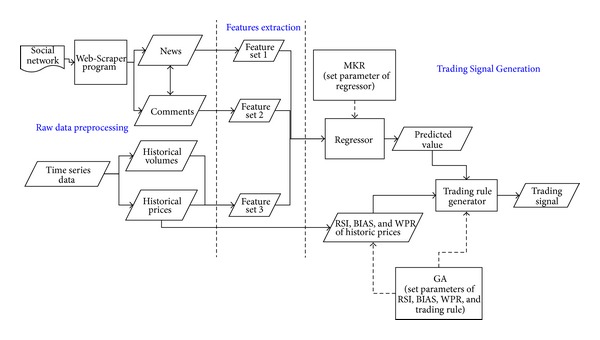
Diagram of the proposed model.

The structure of the proposed model is shown in Figure 1. It is composed of three parts:

- raw data preprocessing,
- features extraction,
- trading signal generation.

### 4.1. Raw Data Preprocessing (RDP)

The RDP part processes the raw data to be used for experiments. For the social network source, the RDP part downloads the news and user comments, including their contents and date of posting, for each day. The frequency of news and user comments and the comment length are calculated by a JAVA program. For the time series data source, the RDP part downloads the historical daily prices (opening, highest, lowest, and closing) and transaction volumes data from the Google Finance website.

### 4.2. Features Extraction (FE)

The FE part extracts the features we need from the data downloaded using the RDP part. For the time series source, the FE part extracts the daily historical opening, highest, lowest, and closing prices and transaction volumes for three famous companies, Amazon, Sony, and Sharp, dealing information technology (IT) products and services. For the social network source, the FE part extracts features of news and user comments (as defined in Section 3.2.2) downloaded using the RDP part.

### 4.3. Trading Signal Generation (TSG)

For the purposes of our study, input feature sets for the proposed model are composed of three parts: (1) features from historical prices and volumes, (2) features from news, and (3) features from user comments. In spite of extensive SVR applications in financial forecasting, SVR models did not address the challenges posed by multiple data sources or multiple representations. In contrast, MKR considers the linear combination of kernels, solves the convex optimization problem of linear combination of kernels, and is guaranteed to achieve the global optimal solution. Hence, theoretically, MKR models perform better than SVR models.

In TSG part, the MKR framework is used to learn a regression function based on these three feature sets and then to predict the stock returns for the next trading day. In experiments, we use one linear kernel and one Gaussian kernel for each input feature set, and we simply set the default values for the parameters of the Gaussian kernel.

The output PC(*t*) of the MKR in the testing period is the predicted stock price change rates at time *t*, which is

(6) $$\begin{array}{c} \text{PC}\left(t\right)=\frac{\text{Predict}\left(t+1\right)-\text{Price}\left(t\right)}{\text{Price}\left(t\right)}, \end{array}$$

where Predict(*t* + 1) is the prediction for stock price for time *t* + 1.

Although the predicted stock price change rates can be used for simulated trading, in our preliminary experiments, the accumulated profits based on just the stock price change rate predictions were not good enough; the same was true for accumulated profits based on using just technical indicators (RSI, WPR, and BIAS). Considering that the prediction and the technical indicators might have complementary components, we propose to combine them to get the trading signal.

After obtaining predictions of the stock price change rate, the proposed method uses a linear function (see (7)) to fuse the predicted stock price change rates and overbought and oversold technical indicators. Parameters of the linear function and technical indicators are learnt by GA. In the GA chromosome design, the accumulated return in the trading period is the fitness function. The TSG part finds a trading rule and executes it to generate a trading signal for the next trading.

The final trading decision value TDV is a linear combination of the overbought/oversold indicators and the stock price change rates predicted by MKR:

(7) $$\begin{array}{c} \text{TDV}=\overset{N}{\underset{i=1}{\sum }}w_{i}e_{i}, \end{array}$$

where *w*_*i*_ are the weights learned by the GA and *e*_*i*_ are the values of the MKR as well as the values of the overbought/oversold technical indicators under consideration (RSI, WPR, and BIAS). Note that the indicators here are expressed as a ratio. We use RSI/100, BIAS, and WPR/100 from Table 1. Further, note that the MKR outputs are stock price change rates that are shown in (6). By these conventions, the *e*_*i*_ in (7) is dimensionless and therefore is consistent.

Once the weights and the other parameters are learned by the GA, we can obtain the decision values TDV on the days for the testing period. In the meanwhile, the threshold for buying (*θ*_buy_) and the threshold for selling (*θ*_sell_) are also learned by the GA. Then, based on the TDV and the threshold values for buying and selling, the trading rule of the proposed method is expressed in Table 5. The trading rule in Table 5 is equivalent to (5). In addition, because the target prediction horizon is one day, our trading rule will simply close the position one day after we open it.

Procedures for the learning of trading rules in TSG part are as follows.

1. Obtain the one-day ahead stock price change rate prediction that was obtained by a regressor trained by MKR.
2. Create chromosomes randomly as the first generation. For every chromosome, apply the trading rules to the training data at every specified time in the training period, by calculating the value of technical indicators, computing the decision value TDV, and making decisions.
3. Compute the accumulated profits during the trading period as the fitness value. Reserve the top 10% of the chromosomes (those that make the top 10% in profit) directly for the next generation. Create new chromosomes using a crossover operation on the chromosomes selected from the current generation (with selection probability based on the fitness score of each chromosome). Repeat the crossover until a new generation is generated. Mutate or flip some bits of the chromosomes randomly.
4. Repeat Steps (2) and (3) until the maximum number (100) of generations is generated or until the fitness of the best individual does not improve for 10 successive generations. Then choose the best chromosome as the one to represent the optimized trading rule. Calculate the return by applying the resulting trading rule on the testing data.

## 5. Experiment Design

### 5.1. Data Sources

Our data for training and testing are from two sources: Google Finance and Engadget. The historical time series data of the daily stock price (opening, closing, highest, and lowest) and daily transaction volumes were obtained from the Google Finance website [29]. We downloaded the attributes pertaining to news items and user comments from the Engadget website [30].

In the experiment, we selected three companies' stocks: Amazon, Sony, and Sharp, which are important companies in US stock markets. Since Engadget is a blog network with daily coverage of gadgets and consumer electronics, it is ideally suited for our purpose.

Because we make daily predictions, the comments and news we use must be published by no later than 9:00 a.m. of day (*T* + 1) to enable us to make a prediction for a said stock. For example, if a news item pertaining to Amazon is published on May 1, then there may be some comments on that news items published by users after 9:00 a.m. on May 2. We cannot utilize such user comments since we attempt a one-day ahead prediction. We use news and user comments data for the three companies from January 1, 2006, to August 15, 2008.

### 5.2. Inputs for MKR

We use data of the preceding week (7 days or (*T* − 6) to *T*) as the input features to predict stock price change rates on the next day, where *T* is the current date or one day before the predicted date. In other words, we assume that the stock price change rates of a company on a certain weekday depend on its stock price and communication activity pertaining to it in the preceding 7 days. We have three sets of input features: features from time series data (historical prices and volumes), features from news, and features from user comments.

#### 5.2.1. Feature Set 1: Features from Trading Data

The first feature set concerns the technical analysis of time series data. We would like to learn to predict changes in stock prices with the MKR framework by using the SMA, MACD, and ROC of the stock price and volume. The details of feature set 1 are shown in Table 6.

#### 5.2.2. Feature Sets 2 and 3: Features from News Dynamics and User Comment Dynamics

Feature sets 2 and 3 consist of features from news dynamics and user comment dynamics, respectively. In this research, we used only numerical features and not, for example, text data. In Table 7, feature numbers 1 and 2 are from news and 3 to 5 are from user comments.

### 5.3. Chromosome Design of the GA

Based on the trading rule design of proposed method, we design the chromosomes (shown in Table 8) for the trading rules that combine signals (described in Section 4.3).

The representations of the genes are as follows.

1. Numbers 1 to 4 (20 bits in total) represent the weights for the three technical indicators and the MKR results. The weight values range from −1 to +1, where the least significant bit represents 2/32 = 0.0625.
2. Numbers 5 and 6 (10 bits in total) represent the threshold values for buying and selling. The range for each threshold is −1 to +1. The least significant bit represents 2/32 = 0.0625.
3. Numbers 7 to 9 (12 bits in total) represent the parameters of RSI, WPR, and BIAS. The values range from 2 to 17.

Before executing the GA training steps, the population size and maximum number of generations are set to 150 and 100, respectively. Individuals are initialized with random chromosomes following the gene structure shown in Table 8. The fitness value is the profit accumulated during the GA learning. In order to retain high-fitness individuals, the elite 10% (the top 10% of individuals in terms of fitness) were reserved automatically at every generation. Therefore, at every generation, the individuals that obtained top 10% of the profits will be reserved for the next generation.

### 5.4. Rolling Window Method for Training and Testing

**Figure 2:**
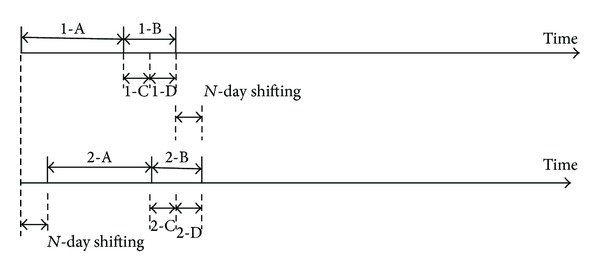
Rolling window method for training and forecasting (for example, 1-A means Period A (see Table 9) in 1st period).

To separate the training and testing periods, we use a rolling window method. We execute the MKR on data of 240 trading days (around 12 months) and obtain the predicted values for the following 160 trading days (around 8 months). The predictions of the first 80 trading days (around 4 months) are used for the GA training and of the remaining 80 trading days are used for the GA testing, that is, to test the whole MKR-GA procedure (see Table 9 and Figure 2). Then, for each subsequent experiment, we move both the training and testing periods forward by 80 trading days (around 4 months). There are, in total, five training and testing periods for each stock from January 1, 2006, to August 15, 2008.

## 6. Models for Comparison and Evaluation Measures

### 6.1. Models for Comparison

We use the term baseline model to designate a simple model implemented easily.  Table 10 lists our proposed model, MKR-all-GA model, and other models for comparison in the experiments including the baseline models. All models (except “Buy and Hold” and “Sell and Hold”) are supposed to output −1, 0, or 1 as trading signal, when the predicted stock price change rate is negative, none, or positive, respectively. If the outputs are predicted stock price change rate, sign function is applied, that is, the function that outputs −1, 0, or 1 if the argument is negative, 0, or positive values. The trading rule is, then, common to these models and simple: when prediction is −1, 0, or 1, a trading agent will sell the stock, wait or buy the stock, respectively, at the start of predefined timeframe, a trading day in the experiments, and close the position at the end of the timeframe.

In Table 10, compared to the proposed model MKR-all-GA, models SVR-ts-GA, SVR-news-GA, and SVR-coms-GA use the SVR as learner with only one input feature set. Model SVR-all-GA uses the same inputs as the proposed model, but it uses SVR as a learner instead of MKR. The following six models (SVR-ts-STS, SVR-news-STS, SVR-coms-STS, SVR-all-STS, MKR-all-STS, and ANN-all-STS) are similar to the first five models, but their trading signal generation rule is simple and fixed and, therefore, is named simple trading strategy (STS): if predicted price change rate is negative, 0, or positive, then −1, 0, or +1 is supposed to output, respectively, which means the trading signal is “sell,” “no trade,” or “buy,” respectively. The two models at the bottom of Table 10 use the same rule with fixed trading position, that is, simply buy or sell the target stocks and wait until the end of the testing period.

### 6.2. Evaluation Measures

To evaluate the goodness of performance of the models, we use the following three measures: root mean square error (RMSE) to evaluate the goodness of fit of models' prediction of price change rates, accumulated return to evaluate the profit-making ability, and Sharpe ratio to evaluate ability to control the risk while yielding good profits.

The RMSE is a frequently used measure of the differences between the values predicted by a model or an estimator and the values actually observed from the entity being modeled or estimated.

In addition, we execute trading based on the trading signals that each model outputs and evaluate the return (loss or profit). In general, high return inevitably accompanies the potential for high risk. Therefore, we attempt to find a method that could decrease risk as well as increase profit. The Sharpe ratio is a measure of the excess return per unit of risk in an investment asset or a trading strategy, named after Sharpe [31].

A summary of these three evaluation measures are shown in Table 11.

## 7. Experimental Results and Discussions

### 7.1. RMSE Results for Stock Price Change Predictions

Every model except for “Buy and Hold” and “Sell and Hold” to be tested has prediction step. In this subsection we evaluate the accuracy of prediction results. Note that the models such as SVR-ts-GA and SVR-ts-STS have the same prediction method so that in this section these two models are summarized as SVR-ts.

Based on the average of RMSE results for the out-of-samples data of the stock price change rate of the three companies (see Table 12), it is obvious that, in the testing periods, the average of RMSEs of the MKR-all for Sharp (0.03117), Amazon (0.01729), and Sony (0.01873) were smaller than those of the other models. It indicates that MKR-based model outperformed SVR-based model in predicting price change rates of stocks. Additionally, at times, the prediction results of SVR-all are not as good as those of methods with just one input: for Amazon, SVR-news (0.03890) and SVR-coms (0.03984) outperform SVR-all (0.04075). Similarly, for Sharp, SVR-news (0.02029) and SVR-coms (0.02251) outperform SVR-all (0.02307); and, for Sony, SVR-news (0.02124) outperforms SVR-all (0.02371). SVR-all also utilizes the same information as MKR-based one, but their prediction results are even worse than some SVR-based models which utilize information from just one source. It indicates that if we use just one kernel, information from different kinds of sources could be harmful although SVM and SVR are thought to be robust to many relevant and irrelevant features.

### 7.2. Profit and Loss Results

Table 13 shows the average returns made by different models in the five testing periods.

First, we note that the results for the models with GA-optimized trading rule, that is, SVR-ts-GA, SVR-news-GA, SVR-coms-GA, SVR-all-GA, and MKR-all-GA, show that most of the average returns (12 of 15 periods) are positive. In Table 13, among the GA-optimized models, the proposed model (MKR-all-GA) yields the best returns for all three stocks, which may be attributed to the fact that MKR-all performed the best in the change rate prediction (in Table 12, we find MKR-all obtains best RMSE results than SVR-ts, SVR-news, SVR-coms, and SVR-all for all three stocks). A comparison of these results evaluated by returns reveals only proposed model, MKR-all-GA, and SVR-coms-GA yielded profit for all three stocks, and if we compare each profit of them, proposed model performed better than the SVR-coms-GA for all three stocks.

Then, we focus on the models with simple trading strategy (SVR-ts-STS, SVR-news-STS, SVR-coms-STS, SVR-all-STS, MKR-all-STS, and ANN-all-STS). We found that while models using the STS could yield good profits for a stock, they also suffered huge losses for other stocks. For example, although SVR-coms-STS yielded an average profit of 16.21% for Sharp, it also suffered an average loss of −7.92% for Sony. In addition, none of the models with the STS made profits for all three stocks.

Finally, we focus on the “Buy and Hold” and the “Sell and Hold” models. For Amazon, “Buy and Hold” yielded an average profit of 15.84%, because its stock price rose significantly during the testing periods. “Sell and Hold” for Sharp yielded an average profit of 3.205%, because its stock price fell a little during the testing periods. However, whether the stock price goes up or goes down is hardly known, which is a starting point of our research on prediction. Neither “Buy and Hold” nor “Sell and Hold” made profits for all three stocks.

### 7.3. Sharpe Ratio Results

We evaluated the Sharpe ratio values of the models for the five testing periods. The interest rate given by the Bank of America for our testing periods is considered as the risk-free return. As the rate ranged between 4% and 5.25% per year, in order to speed up the computation, we assume the risk-free return per year to be 5%. Then, we calculate the average risk-free return of each testing period (4 months) as 1.67%.  Table 14 shows the Sharpe ratios of the returns for each model in the five testing periods.

A higher Sharpe ratio indicates a higher ratio between net return (asset return minus the risk-free return) and volatility. From Table 14, we find that the proposed model obtained positive Sharpe ratio values for all three stocks. Some models yielded better profits than our proposed model for some stocks (such as “Sell and Hold,” SVR-coms-STS, and SVR-all-STS for Sharp). However, the Sharpe ratio values of “Sell and Hold” for Amazon and Sony, of SVR-coms-STS for Sony, and of SVR-all-STS for Amazon and Sony are negative, thus indicating that their average return is less than risk-free return. In addition, SVR-coms-GA and the proposed model (MKR-all-GA) are the only two models with positive Sharpe ratios for all three stocks. Furthermore, on comparing the Sharpe ratios of these two models, we find that the proposed model has a higher Sharpe ratio than SVR-coms-GA for all three stocks. From the results for the average return and the Sharpe ratios, we confirm that the proposed MKL-all-GA model outperforms the baseline and other models in terms of return as well as the Sharpe ratio.

## 8. Conclusions 

In this paper, we proposed a model to generate heuristically optimized trading rules by utilizing social network activities and historical traded prices and transaction volumes. The proposed model extracts three kinds of features from multiple sources. Then it predicts the stock price change rates based on the MKR framework. Finally, GA finds trading rules based on the stock price change rate prediction and three overbought and oversold indicators. We evaluated the prediction and trading performances of the experimental results by RMSEs, accumulated returns, and Sharpe ratio. Experimental results indicate that our proposed model outperforms baseline and other models in stock price change rate prediction, accumulated returns, and Sharpe ratios for three technology companies.

This research is the first of its kind to apply MKR on time series data of prediction target and social network data for a training period. The results show that the prediction of the stock price change rates by MKR was better than that by SVR for all three stocks in terms of RMSEs. We then applied the GA to optimize a change direction predictor that uses the predicted stock price change rate and overbought/oversold indicators in the training periods. We conducted simulated trading of our target stocks and evaluated the results by accumulated returns and Sharpe ratios in the testing periods. From the results in Tables 13 and 14, it is clear that the proposed model outperforms baseline models (“Buy and Hold” and “Sell and Hold”) and other models such as SVR with time series data and SVR with time series data and GA-optimized change direction predictor. Although baseline models and other models outperformed the proposed model in some testing periods, for example, SVR-coms-STS yielded 16.21% for Sharp, only our proposed method obtained good profits (2% to 14.8% profit per testing data set of around 4 months) and consistently positive Sharpe ratios (i.e., 0.23 to 0.38), which is better than SVR-coms-GA which also attained positive Sharpe ratios for the three stocks. In short, our proposed model obtained favorable returns with low volatility over all five testing periods for all stocks in our experiments. This indicates that the proposed model can be used as an effective approach to automatic trading.

## Figures and Tables

**Table 1:** List of technical indicators used in this research.

Indicator	Mathematical formula	Parameters
Simple moving average (SMA)	${\text{SMA}}_{n}(t)=\;\frac{\sum_{k=t-n+1}^{t}\text{Price}(k)}{\;n}$	*n* is the length of timeframe

Exponential moving average (EMA)	EMA_n_(*t*) = Price(*t* − 1)∗*a* + (1 − *a*)∗EMA_*n*_(*t* − 1)	Usually *a* = 2/(*n* + 1), *n* is the length of timeframe

Rate of change (ROC)	$\text{ROC}(t)=\frac{\text{Price}(t)-\;\text{Price}(t-1)}{\text{Price}(t-1)}\;$	

Moving average convergence and divergence (MACD)	MACD(*t*) = EMA_12_(t) − EMA_26_(*t*) , MACD Signal(*t*) = EMA_9_(MACD(*t*))	

BIAS (bias ratio)	${\text{BIAS}}_{n}(t)=100\times \frac{\text{Price}(t)-{\text{SMA}}_{n}(t)}{{\text{SMA}}_{n}(t)}$	*n* is the length of timeframe

WPR (Williams %*R*)	$\%R_{n}(t)=\frac{\text{Price}(t)-{\text{high}}_{n\;\;\text{periods}}}{{\text{high}}_{n\;\;\text{periods}}-{\text{low}}_{n\;\;\text{periods}}}\times 100$	*n* is the length of timeframe

RSI (relative strength index)	${\text{RSI}}_{n}(t)=100-\frac{100}{1+{\text{RS}}_{n}(t)}$	${\text{RS}}_{n}(t)=\frac{\text{Average of positive price changes in }n\text{ days}}{\text{Average of negative price changes in }n\text{ days}}$ ; *n* is the length of timeframe

**Table 2:** List of features from historical traded prices and volumes.

Number	Features based on historical prices and volumes
1	ROC for historical prices
2	ROC for historical transaction volumes
3	SMA for historical prices
4	SMA for historical transaction volumes
5	MACD for historical prices
6	MACD signal for historical prices

**Table 3:** List of numerical features of news items.

Number	Numerical features based on news	Calculation
1	Frequency of news	*f*_*t*_^*c*^
2	SMA of frequency of news	$\text{FNSMA}\left(t\right)=\frac{\sum_{k=t-n+1}^{t}f_{k}^{c}}{n}$ , *n* = 7

**Table 4:** List of features based on user comments.

Number	Features based on user comments	Calculation
1	Frequency of user comments	*F*_*t*_^*c*^

2	SMA of frequency of user comments	$\text{FCSMA}\left(t\right)=\frac{\sum_{k=t-n+1}^{t}F_{k}^{c}}{n}$ , *n* = 7

3	Average and standard deviation of comment length	$a_{t}^{c}=\frac{1}{m}\overset{m}{\underset{k=1}{\sum }}l_{k}^{c}$ , $b_{t}^{c}=\sqrt{\frac{1}{m}\overset{m}{\underset{k=1}{\sum }}{\left(l_{k}^{c}-a_{t}^{c}\right)}^{2}}$

**Table 5:** Trading rule design of proposed model.

Trading rule	Rule in detail
Proposed model	If TDV > *θ*_buy_, the next trading position is “buy”;
	else if TDV < *θ*_sell_, the next trading position is “sell”;
	else (i.e., *θ*_sell_ ≤ TDV ≤ *θ*_buy_) the next trading position is “no trade”.

**Table 6:** Features from stock prices and volumes.

Number	Indicator	On	Description
1	ROC	Closing price	ROC for historical prices from day (*T* − 6) to *T*
2	ROC	Volume	ROC for historical volumes from day (*T* − 6) to *T*
3	SMA	Closing price	SMA for historical prices from day (*T* − 6) to *T*
4	SMA	Volume	SMA for historical volumes from day (*T* − 6) to *T*
5	MACD	Closing price	MACD for historical prices from day (*T* − 6) to *T*
6	MACD signal	Closing price	MACD signal for historical prices from day (*T* − 6) to *T*

**Table 7:** Features from news dynamics and user comment dynamics.

Number	Feature	On	Description
1	Frequency	News	Frequency of news from (*T* − 6) to *T*
2	SMA	News	SMA of news from (*T* − 6) to *T*
3	Frequency	User comments	Frequency of user comments from (*T* − 6) to *T*
4	SMA	User comments	SMA of user comments from (*T* − 6) to *T*
5	MA/standard deviation	User comments	Average and standard deviation of comment length from (*T* − 6) to *T*

**Table 8:** Chromosome design of the GA model.

Number	Length	Value range	Meaning
1	5 bits	−1 to 1	RSI weight
2	5 bits	−1 to 1	WPR weight
3	5 bits	−1 to 1	BIAS weight
4	5 bits	−1 to 1	MKR weight
5	5 bits	−1 to 1	Threshold value for buying
6	5 bits	−1 to 1	Threshold value for selling
7	4 bits	2 to 17	Parameter of RSI
8	4 bits	2 to 17	Parameter of WPR
9	4 bits	2 to 17	Parameter of BIAS

**Table 9:** Training and testing period.

Period	Process	Length of period
A	MKR training	240 trading days (around 12 months)

B	MKR testing (prediction)	160 trading days (around 8 months)

C	GA training	80 trading days (around 4 months)

D	GA testing (trading)	80 trading days (around 4 months)

**Table 10:** A list of models for stock change prediction and trading signal generation.

Model	change rate prediction		Trading signal generation		Description
	Features	Learning method	Features	Learning method	
MKR-all-GA (proposed)	News/comments/historical data	Multiple kernel (MKR)	Prediction/RSI/WPR/BIAS	GA	Integration of MKR and GA. Three feature sets are used in MKR
SVR-ts-GA	Historical data	Single kernel (SVR)	Prediction/RSI/WPR/BIAS	GA	Integration of SVR and GA. Stock prices and transaction volumes are used in SVR
SVR-news-GA	News	Single kernel (SVR)	Prediction/RSI/WPR/BIAS	GA	Integration of SVR and GA. Features from news are used in SVR
SVR-coms-GA	User comments	Single kernel (SVR)	Prediction/RSI/WPR/BIAS	GA	Integration of SVR and GA. Features from user comments are used in SVR
SVR-all-GA	News/comments/historical data	Single kernel (SVR)	Prediction/RSI/WPR/BIAS	GA	Integration of SVR and GA. Three feature sets are used in SVR
SVR-ts-STS	Historical data	Single kernel (SVR)		No learning; sign function	SVR and a simple trading strategy. Features from stock prices and transaction volumes are used in SVR
SVR-news-STS	News	Single kernel (SVR)		No learning; sign function	SVR and a simple trading strategy. Features from news are used in SVR
SVR-coms-STS	User comments	Single kernel (SVR)		No learning; sign function	SVR and a simple trading strategy. Features from user comments are used in SVR
SVR-all-STS	News/comments/historical data	Single kernel (SVR)		No learning; sign function	SVR and a simple trading strategy. Three feature sets are used in SVR
MKR-all-STS	News/comments/historical data	Multiple kernel (MKR)		No learning; sign function	MKR and a simple trading strategy. Three feature sets are used in MKR
ANN-all-STS	News/comments/historical data	Artificial neural network (ANN)		No learning; sign function	ANN and a simple trading strategy. Three feature sets are used in ANN
Buy and Hold	—	No learning		No learning	Buy and then hold the position, until the end of the testing period
Sell and Hold*	—	No learning		No learning	Sell and then hold the position, until the end of the testing period

*Note for “Sell and Hold,” we do margin transaction.

**Table 11:** Summary of evaluation measures.

Evaluation measure	Calculation	Description
RMSE	$\text{RMSE}=\sqrt{\frac{\sum_{i=1}^{n}{\left(\text{PC}(i)-\text{ROC}(i)\right)}^{2}}{n}}$	PC(i) is the predicted price change rate of target stock at time *i*, ROC(i) is the real change rate at time (*i* + 1), and *n* is the number of prediction times.

Accumulated return	$\mathrm{AC}=\overset{m}{\underset{i=1}{\sum }}R_{i}$	*R*_*i*_ is the return in testing period *i*, and *m* is the number of testing periods.

Sharpe ratio	$S=\frac{E\left[R-R_{f}\right]}{\sqrt{\text{var}\left[R-R_{f}\right]}}$	*R* is the asset return, *R*_*f*_ is the return on a benchmark asset, *E*[*R* − *R*_*f*_] is the expected value of the excess of the asset return over the benchmark return, and var[*R* − *R*_*f*_] is the variances of the asset return. In our experiments, we used the Sharpe ratio as an evaluation criterion to evaluate the return-risk ratio performance of the models.

**Table 12:** Average of RMSEs in the five out-of-sample testing periods for three-stock price changes.

Models	Sharp	Amazon	Sony
SVR-ts	0.04756	0.03295	0.03212
SVR-news	0.03890	0.02029	0.02124
SVR-coms	0.03984	0.02251	0.02560
SVR-all	0.04075	0.02307	0.02371
MKR-all	0.03117	0.01729	0.01873

**Table 13:** Average returns in the five out-of-sample testing periods (of four months each) in the ratio of the initial investment.

Models	Sharp	Amazon	Sony
MKR-all-GA (proposed)	0.02143	0.14811	0.03797
SVR-ts-GA	0.01307	0.12478	−0.01702
SVR-news-GA	0.01425	0.08226	−0.02630
SVR-coms-GA	0.01343	0.13386	0.00116
SVR-all-GA	0.01358	0.02097	−0.02479
SVR-ts-STS	0.00399	−0.00258	−0.07979
SVR-news-STS	0.02224	−0.12041	−0.03325
SVR-coms-STS	0.16217	0.13640	−0.07926
SVR-all-STS	0.04633	−0.13022	−0.09349
MKR-all-STS	−0.03845	−0.25684	0.01630
ANN-all-STS	−0.01677	0.17738	0.02611
Buy and Hold	−0.03205	0.15849	0.00543
Sell and Hold	0.03205	−0.15849	−0.00544

**Table 14:** Sharpe ratios in the five testing data sets for the three-stock trading.

Models	Sharp	Amazon	Sony
MKR-all-GA (proposed)	0.23395	0.38152	0.23676
SVR-ts-GA	−0.04203	0.26138	−0.28590
SVR-news-GA	0.15631	0.18533	−0.19119
SVR-coms-GA	0.10704	0.34027	0.00716
SVR-all-GA	0.13337	0.21494	−0.18474
SVR-ts-STS	−0.11036	−0.10034	−1.06153
SVR-news-STS	0.09233	−0.69485	−0.19220
SVR-coms-STS	0.75930	0.23981	−0.45355
SVR-all-STS	0.47225	−0.48811	−0.98314
MKR-all-STS	−0.25124	−1.19644	0.08685
ANN-all-STS	−0.10232	0.56225	0.14567
Buy and Hold	−0.24035	0.41475	0.02929
Sell and Hold	0.24035	−0.41476	−0.02929
